# Hereditary Gingival Fibromatosis: A Review and a Report of a Rare Case

**DOI:** 10.1155/2013/930972

**Published:** 2013-02-21

**Authors:** Hossein Aghili, Mahdjoube Goldani Moghadam

**Affiliations:** Department of Orthodontics, Faculty of Dentistry, Shahid Sadoughi University of Medical Sciences, Yazd 89195/165, Iran

## Abstract

Hereditary gingival fibromatosis (HGF) is a rare condition which manifests itself by an enlarged gingival tissue covering teeth to various extents. The condition may occur isolated or as part of a syndrome. This paper presents a case of 9-year-old female patient suffering from HGF with chief complaint of mouth protrusion. Cephalometric findings showed severe mandibular deficiency and vertical maxillary excess. Patient exhibited perioral muscle contraction on mouth closing. After discussing the treatment possibilities with the patient and her parents, the decision was made to wait until growth potential decreases (following the adolescent growth spurt) and to correct the problem with orthognathic surgery.

## 1. Introduction

Hereditary gingival fibromatosis (HGF) is a rare condition with the prevalence of one per 175000 population and equal distribution in sexes [1]. The onset of disease is concurrent with the eruption of permanent teeth [2]. The excessive growth of gingival tissue may cause displacement of teeth, overretention of primary teeth, and spacing [2]. Both autosomal dominant and autosomal recessive modes of inheritance have been reported [3–5]. The gingival enlargement may occur alone or in combination of other symptoms as part of a syndrome. Examples of syndromic GF are Zimmerman-Laband syndrome (GF, hypoplastic distal phalanges, hepatosplenomegaly, epilepsy, hypertrichosis, and mental retardation), Jones syndrome (GF and progressive neural deafness), Klippel-Trenaunay syndrome (GF, hemihypertrophy, Nevus flammeus, hemangioma, hypertlorism, and macrocephaly), Ramon syndrome (GF, hypertrichosis, mental retardation, epilepsy, rheumatoid arthritis, and diabetes mellitus), Rutherfurd syndrome (GF, unerupted teeth, corneal dystrophy, and mental retardation), and Cross syndrome (GF, nanophthalmos, microcornea, and severe mental retardation) [6]. A case of nonsyndromic HGF with skeletal jaw discrepancy is presented in this paper. 

## 2. Case Presentation

**Figure 1:**
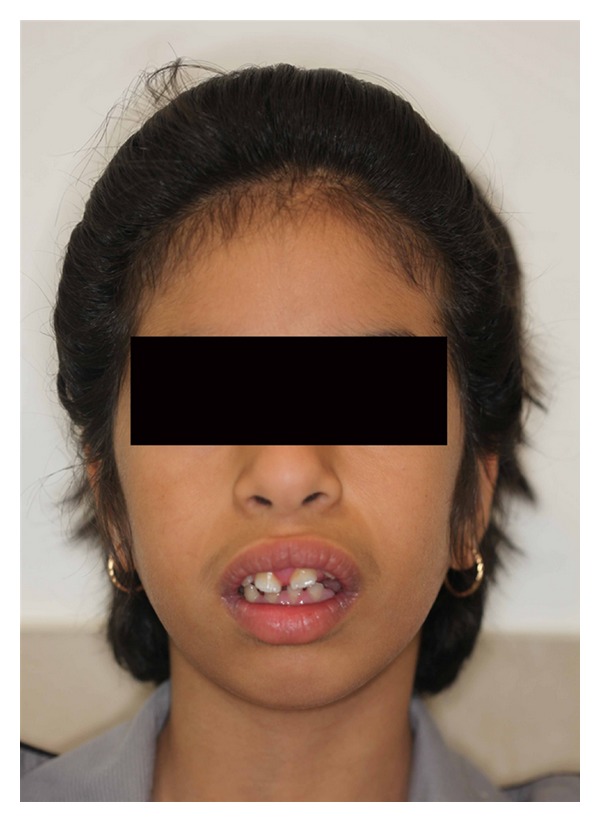
Frontal view of the patient showing lip incompetency and mouth protrusion.

A 9-year-old female patient visited the department of orthodontics complaining of mouth protrusion (Figure 1) and enlargement of gingival tissues. The patient and her parents noticed gingival enlargement when she was seven years old with gradual spacing of teeth. At the time they visited a general dental practitioner who suggested quadrant wise gingivectomy. Patient's dental records represented that maxillary and mandibular quadrant wise gingivectomies with reverse bevel incision had been performed 1 year ago under local anesthesia, but the enlargement has recurred to the significant degree. Patient's medical history was noncontributory and revealed no significant medication. Upon interviewing it was disclosed that the patient's father, sister, and paternal grandfather had a history of similar gingival enlargement. Her intelligence was normal and clinical examination failed to reveal any sign of associated syndromes. A general physician was consulted who confirmed that the condition was an isolated occurrence.

**Figure 2:**
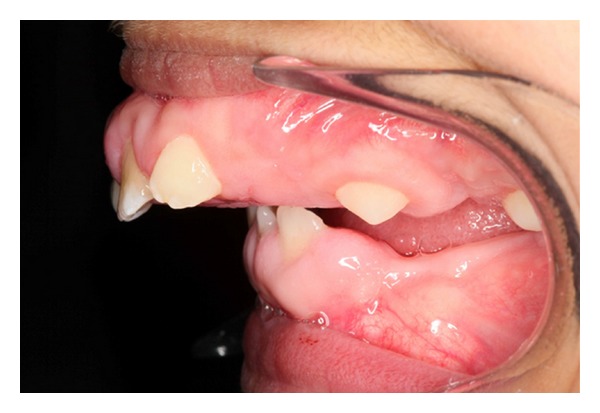
Excessive overjet.

**Figure 3:**
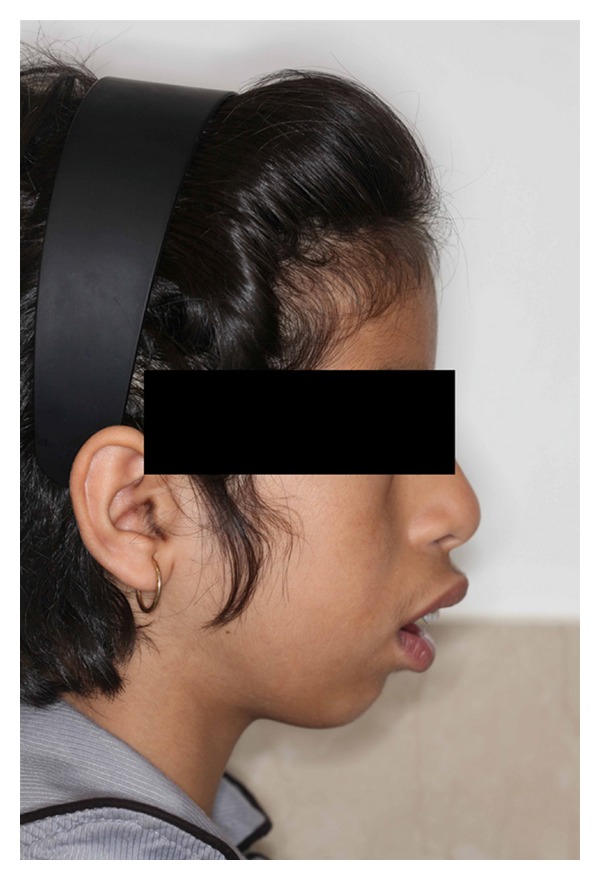
Profile view of the patient showing lip incompetency at rest.

**Figure 4:**
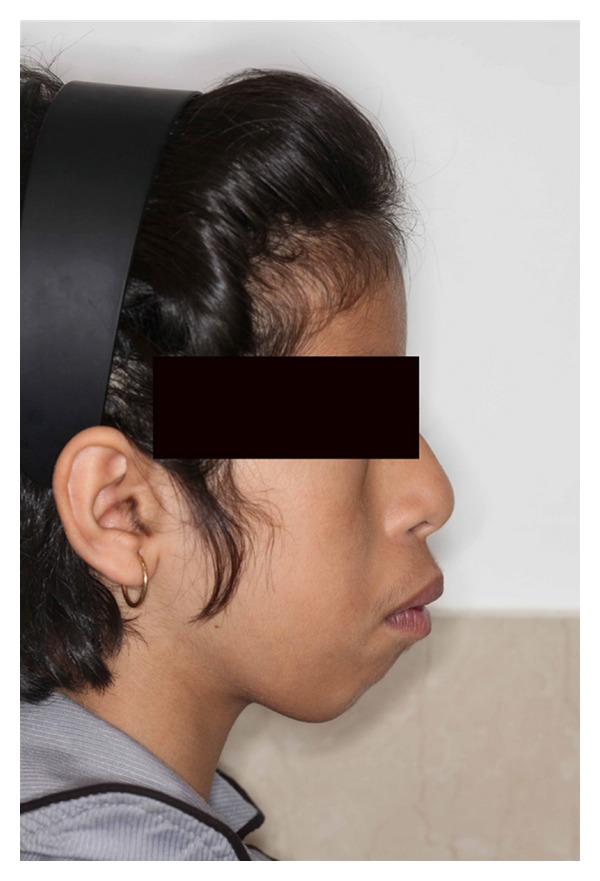
Profile view of the patient showing lip strain.

**Figure 5:**
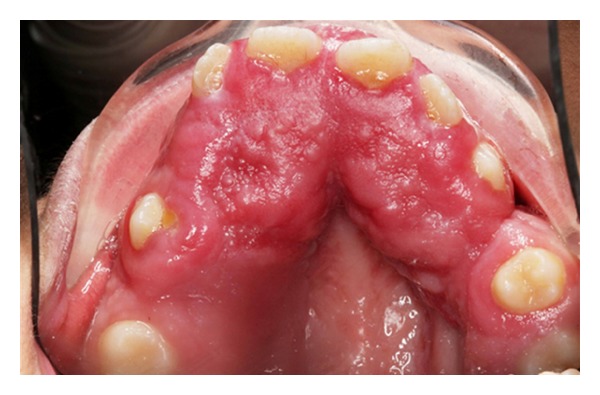
Upper occlusal view.

**Figure 6:**
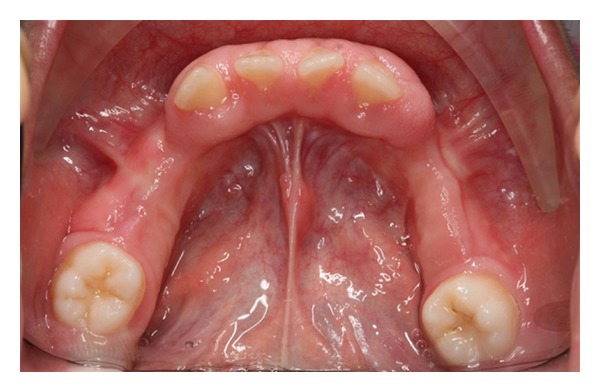
Lower occlusal view.

Intraoral examination showed normal development of the dentition. Excessive overjet and protrusion of upper teeth were observed (Figure 2). The patient showed muscle tension on mouth closing, lip strain and lip incompetency at rest (Figures 3 and 4). The enlarged gingival tissues were normal in color and firm in palpation, and the most affected sites were anterior regions of jaws especially in upper anterior palatal mucosa (Figures 5 and 6).

**Figure 7:**
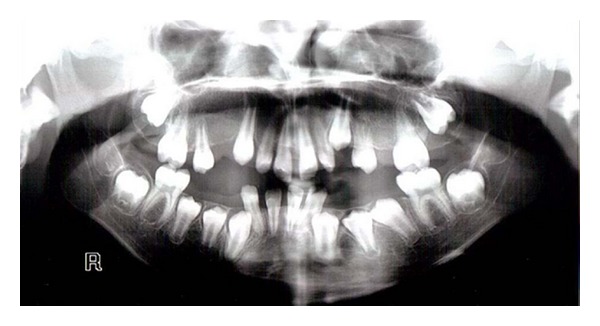
Panoramic radiograph showing normal development of permanent teeth and missing of maxillary right first and left second premolars.

**Figure 8:**
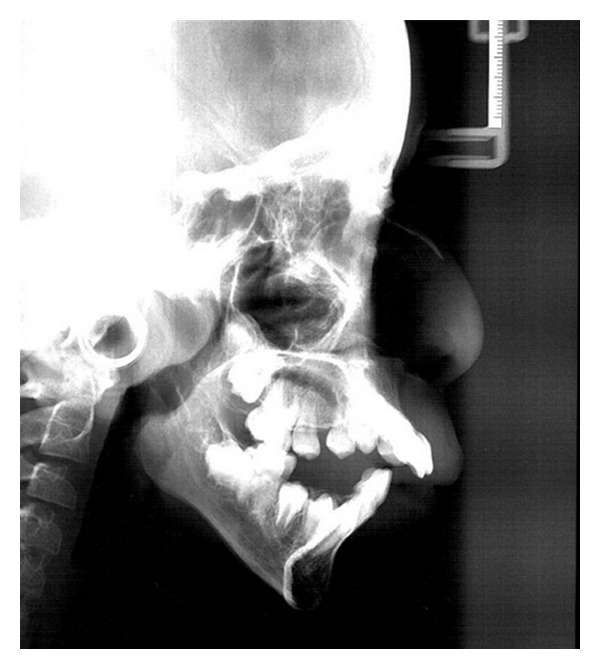
Lateral cephalogram showing mandibular deficiency and vertical growth pattern.

Panoramic radiograph showed normal development of the dentition (Figure 7). Permanent teeth were all present (except for maxillary right first and left second premolars) and in their normal developmental stages. In lateral cephalogram of the patient severe mandibular deficiency and vertical maxillary excess were obvious. Upper anterior teeth were protruded and mandibular plane angle was large (vertical growth pattern) (Figure 8).

Since the dentition showed normal development and the eruption of permanent teeth was not affected by the condition, repeated gingivectomies were not included in the treatment plan. Treatment options were discussed with the patient and her parents, including growth modification (functional appliance combined with high pull headgear) before adolescent growth spurt or orthognathic surgery well after patient's growth spurt at puberty. It was explained for the parents that the long face growth pattern is hard to modify and the treatment must continue over many years which needs excellent cooperation [7]. It also was explained that in a trial on a sample of at least moderately severe Class II patients, the number of patients who needed orthognathic surgery later in life showed insignificant difference between treated children (with growth modification) and control group (no treatment). Regarding the severity of the problem and their unwillingness to attempt growth modification, the decision was made to wait until slowing down the growth (after adolescent growth spurt) and correcting the jaw discrepancy through bimaxillary orthognathic surgery for mandibular advancement and the impact of the posterior maxilla. Until then periodic monitoring of the patient to become assure of normal eruption of permanent teeth was planned. To improve the patient psychological state, it was suggested to temporarily eliminate upper teeth spacing and protrusion by means of a 2 × 4 appliance along with resective periodontal surgery in upper anterior region (vestibular and palatal) which her parents refused. 

## 3. Discussion

The mode of genetic transmission in this patient was consistent with an autosomal dominant inheritance since both sexes and successive generations were affected (the patient, her sister, father and paternal grandfather). As stated earlier both autosomal dominant and recessive modes of transmission of HGF have been reported [3–5]. Recently, four genetically separated loci on chromosome 2p (GINGF and GINGF3), chromosome 5q (GINGF2), and chromosome 11p (GINGF4) have been determined in association with HGF [6, 8–10]. 

Differential diagnosis of gingival enlargements includes enlargement associated with hormonal changes (such as in pregnancy), drug-induced gingival overgrowth, or enlargements due to genetic disorders [11]. Inflammations, tumors, and cysts can also present themselves as gingival enlargements [1]. Enlargement can be further categorized as localized and generalized depending on the extent of involvement [12]. In the present case the diagnosis of HGF was given based on the family history and clinical examination. The enlargement in our patient was particularly prominent in the anterior palate and mandibular anterior region. HGF generally has two forms based on the extent of involved tissue, including nodular (localized, mostly seen in the maxillary tuberosity and molar area) and symmetric (generalized, which is more common and both arches are equally affected) forms [13]. The affected gingival tissue is pink, usually shows exaggerated stippling, and is firm and fibrous on palpation which helps to differentiate the condition from drug-induced gingival enlargements in those where the tissue is often movable [13]. Inflamed gingival tissue due to plaque accumulation may become red. Free and attached gingival tissues are both involved, but extension beyond the mucogingival junction is not observed [13]. 

The histological findings are nonspecific and include acanthotic and hyperkeratotic epithelium with elongated rete ridges deeply extended to a relative avascular connective tissue with densely arranged collagen bundles and numerous fibroblasts [1, 14]. Two types of fibroblasts are present in HGF: inactive fibroblast with little cytoplasm and highly active fibroblast with fully developed cytoplasmic organelles [15]. Activation of certain fibroblasts by physical trauma, such as tooth eruption, can be a possible explanation for simultaneity of HGF development with tooth eruption [16]. 

As stated earlier HGF can be an isolated or syndrome-associated condition. The association of the entity with growth hormone deficiency secondary to lack of growth hormone releasing factor secretion has also been reported [17]. The presented patient seemed to be a case of nonsyndromic HGF which the isolated nature of the condition was further confirmed by a physician.

The side effects of gingival enlargement in HGF include speech problems, painful mastication, spacing and diastema, malocclusion, and overretention of primary teeth [18]. In our patient, gingival enlargement had led to spacing especially between upper anterior teeth. In order to address patients' esthetic and functional needs usually surgical excision of the enlarged tissue is necessary. Since the enlargement recurs to a various extent, repeated gingivectomies are needed for restoring the gingival contours. In the patient presented, deferring orthodontic treatment until growth potential decreases and correcting the problem through combined orthodontic and orthognathic procedures were planned. Gingivectomy had little to do in improving the patient's condition. Regarding the higher rate of recurrence in children and teenagers compared to adults [18], we found it better to delay surgical intervention (gingivectomy) until commencement of the presurgical orthodontic phase and planned periodic visits to monitor eruption of remained permanent teeth. Early treatment with growth modification was rejected by the parents. 

## 4. Conclusion

This paper presents diagnosis and treatment plan of a patient with HGF and severe skeletal malocclusion. Regarding the rarity of the condition only few case reports addressing the orthodontic management of the condition exist. The case presented in this paper differs from previous reports since she needed combined orthodontic and orthognathic treatment because of severe skeletal discrepancies of the jaws. After precise evaluation of risks, costs, and benefits associated with different treatment possibilities and discussing them with the patient and her parents, orthognathic surgery at appropriate time was planned.
